# Whole Genome Sequencing Reveals Virulence Potentials of *Helicobacter pylori* Strain KE21 Isolated from a Kenyan Patient with Gastric Signet Ring Cell Carcinoma

**DOI:** 10.3390/toxins12090556

**Published:** 2020-08-29

**Authors:** Catherine Mwangi, Stephen Njoroge, Evariste Tshibangu-Kabamba, Zahir Moloo, Allan Rajula, Smita Devani, Takashi Matsumoto, Kimang’a Nyerere, Samuel Kariuki, Gunturu Revathi, Yoshio Yamaoka

**Affiliations:** 1 Department of Medical Microbiology, Jomo Kenyatta University of Agriculture and Technology, Nairobi P.O. Box 62000-00200, Kenya; cmwangi99@gmail.com (C.M.); dsznjoroge@gmail.com (S.N.); kinyerere@yahoo.com (K.N.); 2 Department of Medical Microbiology and Parasitology, Kenyatta University, Nairobi P.O. Box 43844-00100, Kenya; 3 Department of Medical Microbiology, Technical University of Kenya, Nairobi P.O. Box 52428, Kenya; 4 Department of Environmental and Preventive Medicine, Faculty of Medicine, Oita University, Oita 879-5593, Japan; evaristetshibangu@gmail.com (E.T.-K.); tmatsumoto9@oita-u.ac.jp (T.M.); 5 Department of Pathology and Laboratory Medicine, Aga Khan Hospital University, Nairobi P.O. Box 37002-00100, Kenya; zahir.moloo@aku.edu (Z.M.); gunturu.revathi@aku.edu (G.R.); 6 Gastroenterology section, Aga Khan Hospital University, Nairobi P.O. Box 37002-00100, Kenya; allan.rajula@aku.edu (A.R.); Smita.Devani@aku.edu (S.D.); 7 Kenya Medical Research Institute, Nairobi P.O. Box 20778-00202, Kenya; skariuki@kemri.org

**Keywords:** *Helicobacter pylori*, Kenya, whole genome sequencing, virulence, virulome, mobilome, Illumina Miseq, Oxford Nanopore MinION

## Abstract

*Helicobacter pylori* (*H.pylori*) infection is etiologically associated with severe diseases including gastric cancer; but its pathogenicity is deeply shaped by the exceptional genomic diversification and geographic variation of the species. The clinical relevance of strains colonizing Africa is still debated. This study aimed to explore genomic features and virulence potentials of *H. pylori* KE21, a typical African strain isolated from a native Kenyan patient diagnosed with a gastric cancer. A high-quality circular genome assembly of 1,648,327 bp (1590 genes) obtained as a hybrid of Illumina Miseq short reads and Oxford Nanopore MinION long reads, clustered within *hpAfrica1* population. This genome revealed a virulome and a mobilome encoding more than hundred features potentiating a successful colonization, persistent infection, and enhanced disease pathogenesis. Furthermore, through an experimental infection of gastric epithelial cell lines, strain KE21 showed the ability to promote interleukin-8 production and to induce cellular alterations resulting from the injection of a functional CagA oncogene protein into the cells. This study shows that strain KE21 is potentially virulent and can trigger oncogenic pathways in gastric epithelial cells. Expended genomic and clinical explorations are required to evaluate the epidemiological importance of *H. pylori* infection and its putative complications in the study population.

## 1. Introduction

*Helicobacter pylori* is a Gram-negative bacillus colonizing half of the world’s population [1,2] and is associated with severe gastro-duodenal diseases, including chronic gastritis, peptic ulcer disease, lymphoma of mucosa-associated lymphoid tissue (MALT lymphoma) and gastric adenocarcinoma [3]. Following several epidemiological studies establishing high incidence of gastric cancer in the *H. pylori*-infected population [4,5], the International Agency for Research on Cancer (IARC) of the World Health Organization (WHO) classified this bacteria as a class I carcinogen in 1994 [6]. Nonetheless, in recent years, the pathogenicity of *H. pylori* has been shown to be deeply shaped by the exceptional genetic diversity and wide geographic variation of the species. This seems to result from the distribution and properties of several bacterial features revealed as virulence factors that grant *H. pylori* clinical strains with abilities to colonize the human stomach, to persist, and to induce diseases [7]. The geographic distribution of these virulence factors has emerged which allows the mapping of the gastric cancer risk worldwide [8].

However, there is persistent incongruence between the prevalence of *H. pylori* infection and the incidence of gastric cancer in several regions [8]. The so-called “African enigma” of *H. pylori* infection has thus been evoked to refer to the contrast observed within African populations between the lowest gastric cancer incidence and the highest *H. pylori* prevalence [9,10]. Several hypotheses related to the human host, to the environment, and to the bacterium have been postulated to clarify this enigma [11,12]. Notably, a lower virulence of African strains has been invoked [7]. However, there is still relatively few studies supporting all these hypotheses [11]. More interestingly, accumulative data suggest that the gastric ulcer and cancer prevalence in Africa might not be as low as reported initially [11,13,14]. It remains disconcerting that the age-standardized incidence rate of gastric cancer in Africa ranges from as high as 20/100,000 in some African regions (i.e., Mali) to as low as 0.3/100,000 in other regions (i.e., Botswana) [13,15,16]. In some African countries such as Kenya, the incidence and mortality rates of gastric cancer are markedly higher than in developed nations such as United States/United Kingdom, yet very scarce published data evaluating etiology, epidemiology, prevention or management exist [13,16]. In this context, studies are needed for circumscribing the epidemiological and clinical relevance of *H. pylori* species colonizing different African regions. These studies have the potential to support current understanding and management of the gastroduodenal disease burden and its distribution in the continent. 

This study is the first to report genomic features and virulence potentials of a *H. pylori* clinical isolate retrieved from a native African patient diagnosed with gastric cancer. The aim is to apply genomic analyses to explore the mobilome and the virulome encoded by the isolate and to experimentally assess its intrinsic ability to trigger gastric epithelial human cells for promoting oncogenic pathways.

## 2. Results

### 2.1. Clinical Outcomes of KE21 Clinical Isolate

**Figure 1:**
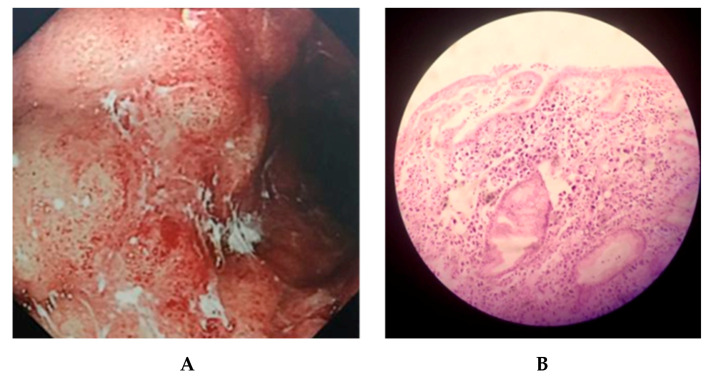
Gastric lesion observed. The panel (**A**) shows endoscopic image obtained by conventional white light imaging in the patient’s stomach. An extended protruding lesion spanning over the greater curvature from the corpus to the antrum of stomach was noted, with irregular and reddened surface, which was bleeding easily on contact. The margin area of the lesion was saw-toothed onto a background mucosa marked with redness. The panel (**B**) shows a histological image obtained by microscopic examination (20×). This fragment of gastric mucosa was lined by dysplastic foveolar type epithelium, with a lamina propria exhibiting a poorly differentiated diffuse neoplasm and signet cells (>50%).

*H. pylori* KE21 was isolated from a gastric biopsy specimen collected by endoscopy from a 61-year-old Kenyan female patient. The patient was referred to the Endoscopy unit at Aga Khan University Hospital Nairobi, for an upper gastrointestinal endoscopy given a history of severe abdominal pain and discomfort. Endoscopic examination identified seemingly non-benign and protruding gastric lesions that were later histologically identified as signet-ring cell carcinoma with depth of invasion in the lamina propria (Figure 1). Further explorations identified a pelvic metastasis of gastric carcinoma. The clinical isolate showed no in vitro resistance to tetracycline (TET) and levofloxacine (LEVO) at Minimum Inhibitory Concentrations (MICs) 0.19 and 0.25 mg/L, respectively. However, resistance was noted to clarithromycin (CLA), amoxicillin (AMX), and metronidazole(MTZ) with MICs of 4 mg/L, 2 mg/L, and ≥256 mg/L, respectively.

### 2.2. Genomic Features of KE21 Clinical Isolate

**Figure 2:**
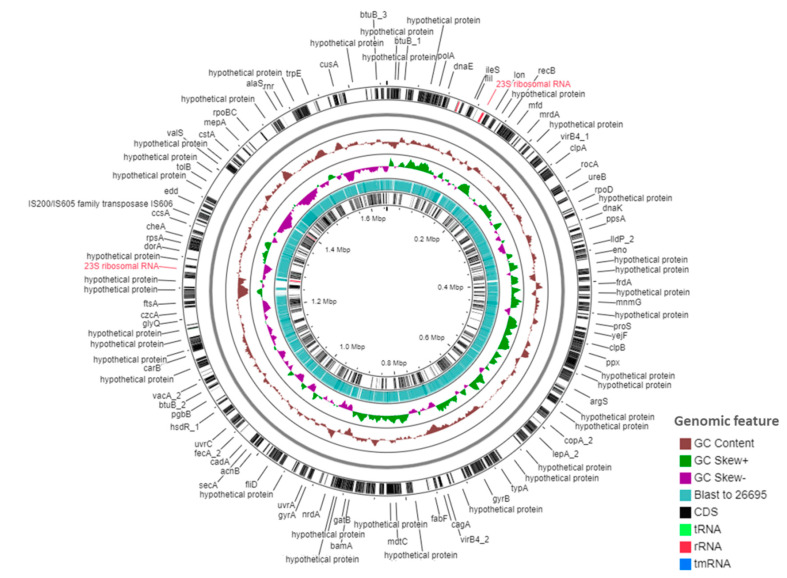
Overview of the genome of *H. pylori* KE21 by circo plot with predicted genes. This circo plot displays an overview of the *H. pylori* KE21 genome as a circular chromosome of 1,648,327 bp length with related information shown onto concentric rings. The outermost and inner rings indicate the genes predicted and annotated by Prokka v. 1.13.3 (Carlton, VIC, Australia, 2018) in the genome. The rings for the GC content, the GC skew, and the BLAST outcomes using *H. pylori* 26695 as reference strain are indicated.

The de novo assembly of the genome of strain KE21 resulted in a single circularized chromosome displayed by one contig sequence of 1,648,327 bp length with a global GC content of 39.1% (Figure 2). Through the CheckM algorithm, the genome assembly reached a completeness of >99% with no detected genetic contamination and strain heterogeneity. The WIMP workflow of Epi2Me [17] assigned 99.9% of MinION reads to *Helicobacter* species. The FastANI [18] used by the DFAST Quality Control tool estimated the highest average nucleotide identity (ANI) of KE21 genome at 94.8% against *H. pylori* CCUG 17874 (GCA_000258845.1). These results conformed the taxonomic assignment of strain KE21 to *H. pylori* species. This genome revealed 1590 predicted genes including 1549 coding sequences (CDSs) through Prokka. Genes encoding thirty-six transfer RNAs (tRNAs) organized into seven clusters and 15 singletons, two separate sets of 5S-23S and 16S ribosomial RNAs (rRNAs), as well as one transfer-messenger RNA (tmRNA) could be identified in the genome. The pan-genome of orthologous genes from strain KE21 and two universal reference genomes, i.e., 26695 and J99, displayed 2508 genes of which 36.5% (916 genes) formed the core genome and 63.5% (1592 genes) formed the accessory genome in the shell (Supplementary Material, Tables S1 and S2). 

#### 2.2.1. Population Genetics of Strain KE21

**Figure 3:**
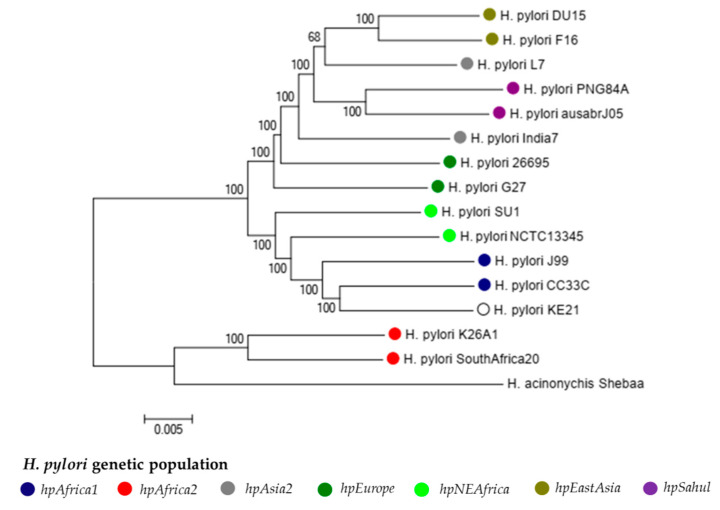
Molecular Phylogenetic analysis of *H. pylori* KE21 with reference strains by Maximum Likelihood method. The neighbor-joining tree was calculated from concatenated nucleic acid sequences of 431 orthologous core genes (length 417,152 bp) between 14 *H. pylori* and one *H. acinonychis*. Each *H. pylori* genetic population and strains within it are indicated. The *H. pylori* KE21 grouped with *hpAfrica1* strains namely J99 and CC33C.

We performed a phylogenetic analysis of strain KE21 with additional 15 representative *Helicobacter* strains (i.e., 26695, J99, SouthAfrica20, CC33CC, NCTC1354, SU1, L7, India7, DU15, F16, G27, K26A1, PNG84A, ausabrJ05, and Shebaa) belonging to existing *H. pylori* genetic populations defined previously by fineSTRUCTURE and STRUCTURE approaches (Supplementary Materials, Table S3) [19,20]. This phylogenetic analysis was accomplished via neighbor-joining tree estimating core genome pairwise distance by Maximum Likelihood method. This analysis clearly classified *H. pylori* genomes into *hpAfrica2*, *hpfrica1*, *hpNEAfrica*, *hpAsia2*, *hpEurope*, and *hpEastAsia* phylogeographic clades. Of note, strain KE21 strain belonged distinctively to the *hpAfrica1* population (Figure 3). 

#### 2.2.2. The Mobilome of Strain KE21

**Figure 4:**
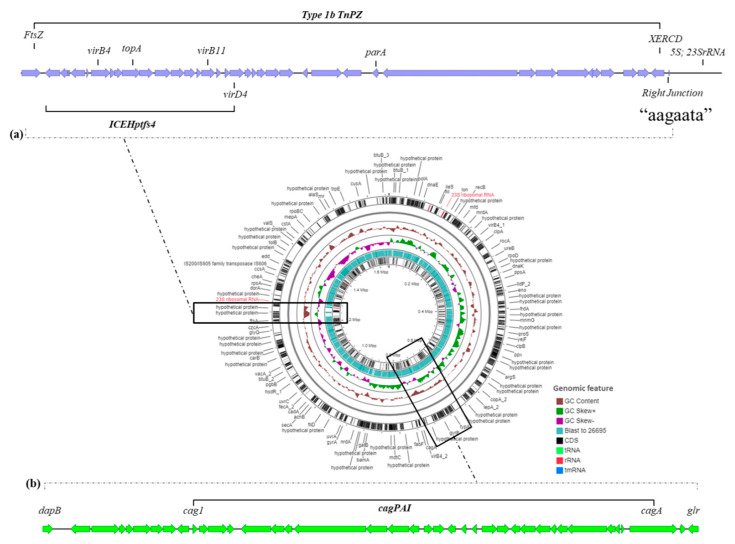
Genomic islands identified in KE21 isolate. This Figure depicts two genomic islands (GIs) through the *H. pylori* KE21, with substantially lower GC content (average ~ 35.6%) compared to the remaining genome (average ~ 39.1%). (**a**) The Transposon Plasticity Zone (TnPZ), delimited by genes of putative FtsZ and 5S-23S rRNAs, and encoding a cluster of vir homolog genes for an integrated conjugative element of *H. pylori* type 4 secretion system of type 4 (ICEH*ptfs*4). (**b**) The dg-region, delimited by genes encoding the 4-hydroxy-tetrahydrodipicolinate reductase (*dapB*) and the glutamate racemase (*glr*) and described, and encoding a *cag* pathogenicity island (cagPAI) region.

No prophage or plasmid could be detected in strain KE21 by using PlasmidSeeker [21] and PHASTER [22] while two genomic islands with an average lower GC content of 35.6% were identified (Figure 4). The first island comprised 46 predicted CDS spanning over 40,235 bp (coordinates: 1221910-1262145 in genome sequence) and was located between genes encoding putative FtsZ and 5S-23S rRNAs, an insertion site described previously as a “transposon, plasticity zone” (TnPZ). As shown in Figure 4, the organization of this TnPZ was typically of type 1b including a cluster of adjacent *vir* homologue genes encoding an integrating conjugative element type four secretion system of type 4 (ICEH*ptfs*4) [23,24]. However, this KE21 ICEH*ptfs*4 cannot be assigned to any of the known subtypes (Supplementary Materials, Table S4) [23]. The second genomic island had 47 predicted CDS that were inserted within a chromosomal region of 49,454 bp (coordinates: 706,360-755,813 in genome sequence) located between genes encoding the 4-hydroxy-tetrahydrodipicolinate reductase (*dapB*) and the glutamate racemase (*glr*), a region known as dg-region [25]. Remarkably, this dg-region encoded a cluster of cytotoxin-associated genes known as *cag* pathogenicity island (cagPAI) (Supplementary Materials, Table S5). 

#### 2.2.3. The Virulome of Strain KE21 

**Figure 5:**
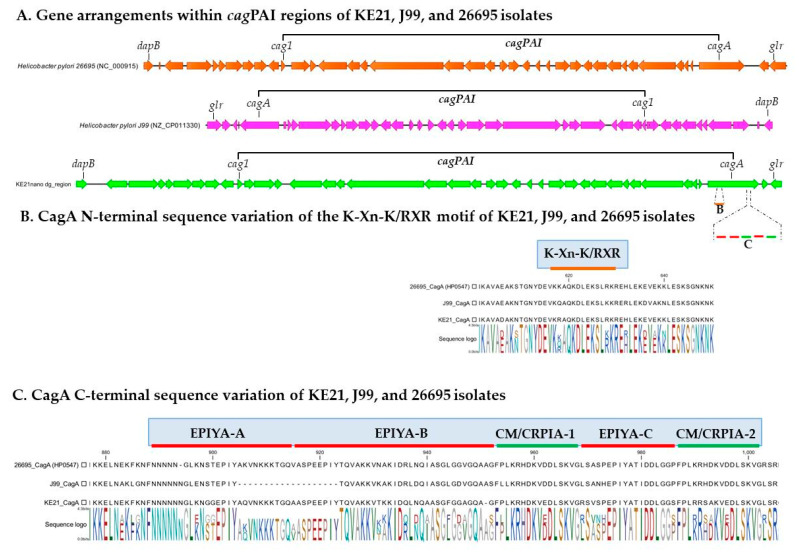
Gene arrangements within the *cag* pathogenecity island (cagPAI) and CagA sequence variations of strain KE21. This Figure depicts the genes arrangement withing the cagPAI of *H. pylori* KE21 in comparison to those in J99 and 26695 reference strains (**A**). The genes are drawn as filled arrows. Analysis of sequence variations of CagA indicates that strain KE21 has a well conserved K-Xn-X/RXR motif of its plecstrin homology (PH) domain (**B**). The C-terminal CagA has three EPIYA segments of type ABC and two CagA-multimerization motifs (CM or CRPIA motif) of type FPLKRHDKVEDLSKVG and FPLKRRSAKVEDLSKVG which stretch proximal and distal to the EPIYA-C, respectively (**C**).

**Figure 6:**
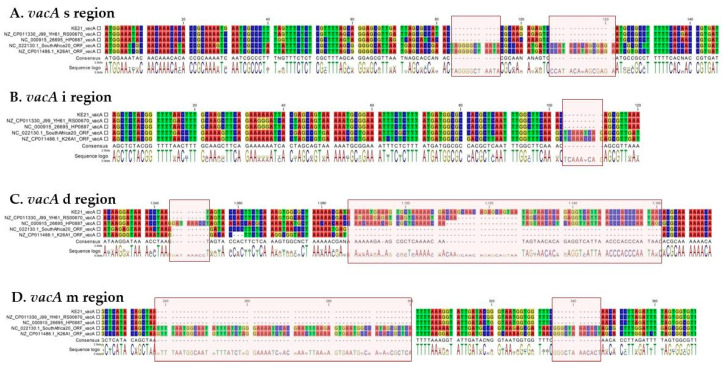
Characterization of *vacA* gene of strain KE21. This Figure displays aligned *vacA* sequences of *H. pylori* KE21, J99, 26695, SouthAfrica20, and K26A1. The sequences shown focus specifically on the four main regions of diversity in *vacA* sequences that have been recognized, namely the signal sequence region or s-region (**A**), the intermediate region or i-region (**B**), the deletion region or *d*-*region* (**C**)**,** and the middle region or (m)-region (**D**) [37,38,39]. The *vacA* alleles characterized by deletions in *s*-, *i*-, and *m*-regions correspond to *s1*, *i1*, and *m1,* respectively; otherwise they are classified as to *s2*, *i2*, and *m2*. In contrast, the *vacA* allele with a large deletion in *d*-region corresponds to *d2* type; otherwise it is classified as *d1* type. Multiple combinations of *s*-, *i*-, *d*-, and *m*-region types may be observed [37,39]. Of note, the strain KE21 *vacA* is of *s1i1d1m1* allele.

By using ABRicate to screen the genome of *H. pylori* KE21 against a customized virulome database, we identified 147 genes that encode proteins whose function relates basically to motility, chemotaxis, cell-to-cell adherence, persistence, acid resistance, and host tissue damage (Supplementary Material, Table S6). First, we noted a cagPAI region of 36,507 bp (coordinates: 717,715–754,221) within a dg-region whose synteny and organization had been previously described as being of type-A (Figure 5A) [25]. A total of 28 CDSs were predicted in this cagPAI including genes of all known components of a syringe-like type four secretion system (T4SS)—i.e., *cagζ/cag1, cagε/cag2, cagδ/cag3, cagγ/cag4, cagβ/cag5, cagα, cagZ, cagY, cagX, cagW, cagV, cagU, cagT, cagS, cagQ, cagP, cagM, cagN, cagL, cagI, cagH, cagG, cagF, cagE, cagD, cagC*, and *cagB*—as well as *cagA* which represent major virulence factors with a well-acknowledged causative role in gastric cancer development [26,27]. The *cagA* gene-encoded CagA oncogene protein of *H. pylori* KE21, intended for delivery into gastric epithelial cells via the T4SS machinery, could be predicted (Figure 5). Its N-terminal sequence showed a plecstrin homology (PH) domain with a conserved K-Xn-X/RXR motif which is required for the bound of CagA to host membrane phosphatidylserine (PS) as a prerequisite for pathophysiological activities of the oncoprotein in polarized epithelial cells (Figure 5B) [28]. Furthermore, the C-terminal CagA repeat sequences comprised three Glu-Pro-IleTyr-Ala (EPIYA) motifs within segments of type ABC, previously referred to as of Western-type, which may undergo tyrosine phosphorylation to hijack carcinogenic intracellular pathways (Figure 5C) [27]. Interestingly, proximal and distal to the 34-amino-acid of the EPIYA-C segment, we identified two variants of CagA-multimerization motifs (yet to be described) also known as conserved repeat responsible for phosphorylation-independent activity motifs (CM or CRPIA motifs)—i.e., FPLKRHDKVEDLSKVG and FPLKRRSAKVEDLSKVG. Second, we identified a vacuolating activity associated gene A (*vacA*) that encodes VacA protein which is a pore-forming toxin of *H. pylori* species with pleiotropic biological effects [29]. Allelic variations of the *H. pylori* KE21 *vacA* was typically of type s1i1d1m1, a genotype associated with increased toxicity, gastric inflammation, peptic ulcer, and gastric cancer development in previous studies (Figure 6) [29,30]. Third, genes encoding more 54 putative adhesins for *H. pylori* KE21 interaction with surface receptors on gastric epithelial cells could be detected [31]. These include main outer membrane proteins (OMPs)—e.g., BabA (HopS), OipA (HopH), HopQ (Omp27), HomA, AlpA (HopC), AlpB (HopB), SabB (HopO) and SabA (HopP)—that are critical in the pathogenesis of *H. pylori* infection [31,32,33]. The characteristics of these genes are shown in Table 1 and in Supplementary Materials, Table S6 and Figures S1 and S2. Some of the outer membrane proteins (OMPs) were encoded by duplicated gene copies (e.g., *babA, homA*). In contrast, genes encoding few putative virulence factors (i.e., BabC or HopU, BabB or HopT, IceA, and DupA) were not detected in KE21 genome (Table 1 and Supplementary Materials, Table S6). Furthermore, a cluster of all seven urease genes (i.e., *ureA/B, ureI*, and *ureE-H*) that are required for resistance and survival in the harsh acidic environment of the stomach was detected in KE21 genome [34]. Finally, the isolate was also equipped with several genes encoding flagella components (e.g., *flgE, flaA*, and *flaB*) and lipopolysaccharides (e.g., *rfaJ, rfaC*) mediating bacterial motility and immune modulation while contributing to *H. pylori* virulence as putative bacterial endotoxins (Supplementary Materials, Table S6) [35,36]. Overall, the genetic features described above reveal potentials for successful colonization, persistent infection, and disease pathogenesis during infection with *H. pylori* KE21. 

### 2.3. Experimental Virulence Assays on KE21 

**Figure 7:**
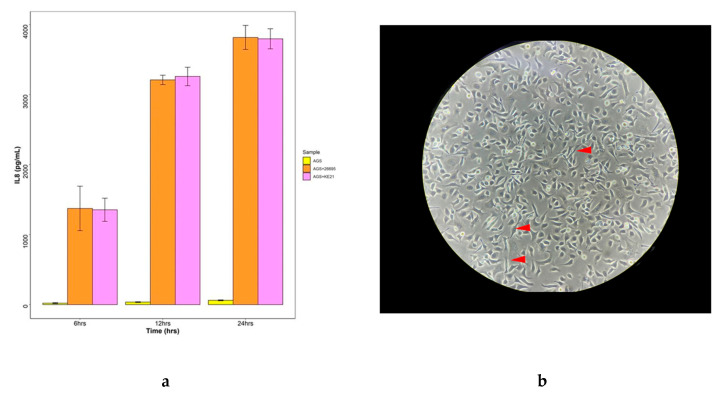
*H. pylori* KE21-induced IL-8 secretion and hummingbird phenotype in gastric epithelial cells line. The panel (**a**) represents the induction of IL8 during infections of gastric epithelial AGS cell lines. AGS cells were incubated with or without various *H. pylori* KE21 or *H. pylori* 26695 (multiplicity of infection 50) for 6, 12 and 24 hrs. IL-8 secretion in the culture supernatant was measured via ELISA. Data are presented as the mean ± standard deviation of three separate experiments. AGS treated with either *H. pylori* KE21 or *H. pylori* 26695 induced high levels of IL8 compared with the untreated one. The panel (**b**) shows a microscopic field of human gastric epithelial AGS cells co-infected with *H*. *pylori* KE21 for 12 hrs. Red arrows point out some cells with induced hummingbird phenotype.

To further assess the virulence abilities of *H. pylori* KE21, we conducted an in vitro infection experiment using the AGS epithelial cells line. Consistently with an intact and functional cagPAI-related T4SS [26,27], KE21 showed ability to promote interleukin-8 (IL-8) production and to produce morphological changes called hummingbird phenotype in AGS cells. Furthermore, a phosphorylated CagA was detected in AGS cells, attesting the competence of KE21-related T4SS for translocation of a bioactive oncoprotein in human epithelial cells (Figure 7). 

## 3. Discussion

*H. pylori* species is characterized by an exceptionally high genetic diversity and geographic variability driving substantial difference in clinical outcomes between different regions. Despite its well-acknowledged causative role in severe gastrointestinal diseases including peptic ulcers and gastric adenocarcinoma; the clinical relevance of African *H. pylori* strains is still debated given the reported contrast between the highest prevalence of the infection and the lowest incidence of gastric cancer in the continent. This discrepant epidemiological situation had been referred to as the so-called “African enigma” [14]. This study is the first to describe genomic features and virulence potential of typical African *H. pylori* isolate retrieved from a native African patient with gastric cancer. Through the genome of isolate KE21, we screened the expanded virulome of *H. pylori* species with the aim to depict its potentialities to promote gastric carcinogenesis in general and signet-ring cell carcinoma (SRCC) in particular. We thus detected tens of genes encoding factors involved in cell-to-cell adherence, acid resistance factors, cell motility and chemotaxis, immune response evasion, as well as in direct tissue damages. An experimental infection of gastric epithelial cells demonstrated the ability of strain KE21 to induce carcinogenic signals. While this report cannot establish a causality link between the isolate and the diagnosed gastric cancer in our patient, the discussion made grasps the full scale of the clinical threat this African *H. pylori* strain would represent during the infection. 

It is notable that this clinical *H. pylori* strain (KE21) was isolated from a Kenyan patient diagnosed with gastric SRCC. SRCC is a unique type of gastric cancer classified as diffuse type (in contrast to intestinal type) according to Lauren’s classification, given its poorly differentiated histological aspect with the lack of the intercellular adhesion and presence of scattered cells of signet-ring morphology predisposed to diffuse invasion throughout the stroma [40,41]. This cancer is found in 8 to 30% of gastric cancers, has unfavorable prognosis while affecting more frequently women from 55 to 61 years old, consistently around 7 years younger than non-SRCC gastric cancer cases [40,42,43]. Consistently with this general profile of SRCC cases, our patient was a 61-year-old female. In contrast with the pathogenicity of intestinal-type gastric cancer that follows the Correa’s cascade consensually linked with *H. pylori* infection, the development of diffuse-type gastric cancer remains mostly elusive and controversial [42]. The SRCC is widely believed to arise from distinct biologic pathways involving genetic abnormalities in the host such as alterations of cell adherence factors like E-cadherin [40,42]. However, increasing epidemiological data have been also associating *H. pylori* infection with sporadic diffuse-type gastric cancers likely through carcinogenic pathways that are independent from gastric mucosa atrophy [44,45,46,47,48,49]. Several pathways probably exploited by the *H. pylori* to induce SRCC-like abnormalities in gastric epithelial cells have been reviewed recently [42]. Consequently, the etiological role of *H. pylori* in both intestinal-type and sporadic diffuse-type gastric cancer is currently plausible. We attempted to explore the genomic attributes of strain KE21 in light with different pathways able to trigger carcinogenic development and *H. pylori*-related pathogenesis in general.

Overall, the metric characteristics of the genome of strain KE21 were consistent with genomic features (e.g., size, structure, gene content) usually reported in non-African isolates [50]. This genome included a mobilome comprising no prophage or plasmid but two genomic islands (GEIs) inserted within regions previously identified as the “transposon, plasticity zone” (TnPZ) and the “*dg*-region” [23,24]. GEIs are syntenic blocks of genes acquired horizontally and that likely contribute to the diversification and adaptation of microorganisms, thus having a significant impact on the genome plasticity and evolution [51]. A close analysis of the KE21 TnPZ assigned its structure to the type 1b and identified an integrating conjugative element type four secretion system of type 4 (ICE*Hptfs*4) commonly occurring in *H. pylori* species [23,24]. TnPZs and ICE*Hptfs*(s) are highly conserved in *H. pylori* while displaying great allelic diversity. Excluding mosaic and remnant forms, TnPZ have been structurally categorized based on their gene arrangement in three types: 1, 1b, and 2; while ICE*Hptfs(s)* have been grouped into two types with related subgroups: ICE*Hptfs*3 and ICE*Hptfs*4 (i.e., ICE*Hptfs*4a, 4b, and 4c) [23,24]. Interestingly, the ICE*Hptfs*4 identified in strain KE21 could not be assigned to any of the known subtypes, suggesting being a new ICE*Hptfs*4 allele. The T4SSs contained in ICE*Hptfs(s)* have been shown to contribute to bacterial virulence through both epidemiological and in vitro infection model studies [52]. However, further studies are still needed to completely elucidate the structure and function of these ICE*Hptfs(s)/*T4SSs as well as their possible interactions with other bacterial virulence factors [52]. In contrast, the content of the *dg*-region, delimited by the *dapB* and *glr* genes, has been extensively explored [25]. Depending on the strain, the *dg*-region may carry a 40-kb DNA segment known as the *cag* PAI region which generally consists of 26 or 27 genes encoding a special syringe-like structure T4SS and the oncoprotein CagA, two major virulence factors in *H. pylori* species [53]. The *cag* PAI region is thought to have been introduced into the *H*. *pylori* genome via horizontal transfer from an unknown source. This island has been show as being prone to functional disruption due to various genetic rearrangements occurring within and outside the constituent genes [25,54]. The intactness or rearrangement of the *cag*-PAI has therefore been thought to be crucial for the progression of gastroduodenal pathology due to *H. pylori* [25,55]. Notably, we found that the *dg*-region of strain KE21 comprises an intact type-A *cag* PAI region, encoding a complete T4SS with a CagA oncoprotein and whose rearrangement is compatible with a biological functionality. 

Among the various virulence factors of *H*. *pylori*, the oncoprotein CagA plays a central role as a scaffolding protein in the development of gastric cancer [54,56,57]. The CagA is a cellular effector whose injection into host cells by the *cag* PAI T4SS deregulates an impressive number of molecular signaling processes including carcinogenic pathways [54]. Some of these pathways involve the binding of the non-phosphorylated CagA to E-cadherin that ultimately trans-activates the β-catenin-dependent genes while inducing also mutational alterations (e.g., TP53) as well as aberrant DNA hypermethylation and inactivation of the CDH1 gene associated with the progression of sporadic diffuse-type gastric cancers [58,59,60,61,62]. Otherwise, the translocated CagA may also undergo a phosphorylation by host cell kinases at a conserved tyrosine residue found within the EPIYA (Glu-Pro-Ile-Tyr-Ala) motif. This allows binding the phosphorylated CagA to a SH2-domain-containing protein tyrosine phosphatase (SHP2), and thus deregulates the phosphatase activity of SHP2, a crucial step in the development of *H. pylori*-related intestinal-type gastric cancer [54,56,63]. The structure of the CagA oncoprotein is crucial for the virulence of *H. pylori* and leads to pathogenic differences. We observed that the KE21 strain comprises in its N-terminal CagA, a cluster of conserved basic residues, known as the basic patch or K-Xn-X/RXR motif, that plays an important role in the interaction of CagA with phosphatidylserine required for the biological activity of *cagA* [28]. In addition, the CagA C-terminal tail of KE21 is characterized by the presence of three EPIYA (Glu-ProIle-Tyr-Ala) motifs, which serve physiologically as motifs for tyrosine phosphorylation of T4SS-delivered CagA by host cell kinases such as Src-family kinases (SFKs) and c-Abl [56]. Based on the sequences flanking each of the EPIYA motifs, we concluded that the KE21 CagA was of ABC type which is termed Western CagA, as it was identified first in Western countries, in contrast to the East Asian CagA of ABD type [57]. The Western type CagA ABC is known to be competent for tyrosine phosphorylation and able to even undergo a sequence amplification of EPIYA-C motifs to more efficiently bind to SHP-2 for increased carcinogenicity [54,57]. Furthermore, the C-terminal tail of the strain KE21 CagA contains another repeatable sequence motif, originally designated as the CagA multimerization sequence motif (CM) comprising 16 amino-acid residues and located immediately distal or proximal to the last repeat of the EPIYA segments [57,64]. Whereas the CM motif sequence is highly conserved, there are several variants previously described in East Asian (CM^E^ type, FPL**R**RS**AA**V**N**DLSKVG), Western (CM^W^ type, FPLKRHDKVDDLSKVG), and Amerindian *H. pylori* species (CM^AmI^ and CM^AmII^ types, **SS**LKRH**A**KVDDLSKVG and **YT**LK**M**H**AGD**D**N**L**R**SKVG) [65,66,67]. Aberrant pro-oncogenic signals elicited by deregulated SHP2 via the EPIYA motif, together with destruction of the gastric epithelium caused by CM-mediated PAR1 inhibition are two major pathophysiological processes that cooperatively contribute to *H. pylori* CagA-induced gastric oncogenesis [57]. Remarkably, we observed that the KE21 CM motifs—i.e., F**P**LKRHDKV**E**DLSKVG and FPLK**R**R**SA**KV**E**DLSKVG—are different from the motifs described previously in non-African populations. However, while the KE21-CagA contains two motifs as in Western type CagA, its most distal motif is very similar to East Asian type (with only two amino acid differences) which binds the most strongly to PAR1 with enhanced biological effect [57]. Given these similarities with CM^E^ and CM^W^ types, pending further studies, we hypothesize that the KE21 CM motif is biologically functional in contrast to Amerindian CM variants (i.e., CM^AmI^ and CM^AmII^) that had been shown as abrogating the ability of CagA to interact with PAR1 and substantially attenuating the CagA oncogenicity [65,66]. Further analyses exploiting an in vitro infection model using the AGS epithelial cells line, indirectly attested the competence of KE21-related T4SS for translocate a bioactive CagA oncoprotein [26,27]. Hence, *H. pylori* KE21 demonstrated ability to promote the production of the proinflammatory cytokine IL-8 from gastric epithelial cells which represent hallmarks of *cag* PAI/T4SS function [54,56]. Moreover, showed the capacity to produce morphological changes of gastric epithelial cells, referred to as “hummingbird phenotype” of AGS cells, which reflect CagA-induced carcinogenic signaling pathways resulting in cytoskeletal rearrangement, cellular motility, and elongated shape of host cells [54,56]. We noted also that the strain KE21 genome encodes a full-length gene for the high temperature requirement A (HtrA) protein. Ubiquitously, *H. pylori* expresses HtrA, a protein with dual function acting as a chaperone and a serine protease, which cleaves-off the ectodomain of E-cadherin and disrupts intercellular adhesions opening up the intercellular space for transmigration of bacteria [68]. Consequently, HtrA-dependent E-cadherin shedding strongly enhances CagA delivery into infected host cells via integrin β1 essential for gastric cancer development [69]. Furthermore, strain KE21 was also found with a full-length gene for the VacA, a key and ubiquitous toxin for pathogenesis in *H. pylori* species [30]. The VacA toxin is known for its multitude of effects on epithelial cells, varying from endosomal alterations of intraluminal pH and disruption of endocytic compartment trafficking, induction of autophagy and enhancement of mitochondrial dysfunction, which can result either from its pore-forming ability or through the activation of pro-apoptotic factors [38,39]. Four main regions of diversity in VacA sequences have been recognized, namely the signal sequence region (*s*)-region, the intermediate region (*i*)-region, the deletion (*d*)-region**,** and middle region (*m*)-region [37,38,39]. These result in VacA alleles containing multiple combinations of s-, i-, d-, and m-region types [37,39]. Being of the *s1i1d1m1* allele, the KE21 VacA is thus a variant that has been reported with enhanced vacuolating activity, and linked to a potentially higher relative risk for development of gastric cancer or peptic ulcer disease [39]. 

Hence, the analysis of cagPAI/CagA, HtrA, and VacA clearly raises the potential for strain KE21 to cause tissue damage and to trigger carcinogenic pathways in epithelial cells. To explore further the full virulence potential of strain KE21, we screened the genome for the presence of genes encoding other factors that are critical in different steps of *H. pylori* colonization and pathogenesis. We thus observed that strain KE21 encodes a cluster of all seven urease genes (i.e., *ureA/B, ureI,* and *ureE-H*) whose activity is required for adjusting the periplasmic pH as an acid acclimation mechanism to resist and survive in the harsh acidic environment of the stomach [34]. The isolate is also equipped with several genes encoding flagella components (e.g., *flgE, flaA*, and *flaB*) and lipopolysaccharides (e.g., *rfaJ, rfaC*) mediating bacterial motility and immune modulation enabling colonization and persistence in the stomach [35,36]. The bacterial attachment to the epithelial cells, as an important step of the infection, is mediated by an impressive number of adhesins and OMPs in *H. pylori* species [32]. We identified more than 50 genes encoding putative OMPs including main proteins that have been formally implicated in the pathogenesis of *H. pylori* infection, e.g., BabA (HopS), OipA (HopH), HopQ (Omp27), HomA, AlpA (HopC), AlpB (HopB), SabB (HopO) and SabA (HopP) [32,33]. However, few putative virulence factors including BabC (HopU), BabB (HopT), IceA, and DupA were not detected in the KE21 genome. BabA, the best characterized of the adhesin proteins in *H. pylori*, mediates binding to host cells’ fucosylated Lewis b (Le(b)) blood group antigens and was encoded in two copies likely granting strain KE21 interesting potentials for host-bacterium interactions [70]. OipA may serve as an adhesin altering the host immune response but also promotes inflammation was predicted to be preserved in switched “ON” phenotype in strain KE21, suggesting preservation of functions [71]. The HopQ outer-membrane adhesin of *H. pylori* exhibits a high level of genetic diversity, and two families of HopQ alleles have been described (type I HopQ and type II HopQ) [72]. The strain KE21 displays HopQ gene encoding type I allele which has been documented to be present in *cag* PAI positive *H. pylori* strains and is epidemiologically associated with gastric cancer [73,74]. This protein was recently shown to bind carcinoembryonic antigen-related cell adhesion molecules (CEACAMs) including CEACAM1, an inhibitory receptor expressed mainly by activated T and NK cells and involved in cancer development and progression [73,75,76]. 

To cope with the need to attest the African origin of the strain, we performed phylogenetic analyses that assigned the isolate to *hpAfrica1*, a major genetic population of *H. pylori* species that is native to Africa [19,20]. *H. pylori* species is split into distinct bacterial populations exhibiting tight relationships with ethno-geographical distribution and history of human host [3,77]. Of these populations, three are originating from Africa (*hpNEAfrica*, *hpAfrica1* and *hpAfrica2*), one from Europe (*hpEurope*), and three from Asia (*hpEAsia*, *hpAsia2* and *hpSahul*) [3,77,78,79]. Our results suggest strongly that strain KE21 was probably not imported from outside Africa. Nevertheless, reporting on a single isolate which may not be representative of all the strains circulating in the Kenyan population constitutes the main limitation of this study. Furthermore, the experimental analyses used in this study constitute a model which only partially reflects conditions in vivo. In vivo studies, using for example animal models, would have further strengthened the validity of our observations. Obviously, further studies are needed to enhance our observation and to fully understand the epidemiological threat and the clinical implications that would result from isolates displaying similar biological properties as strain KE21 that probably are spreading in the study population. 

## 4. Conclusions

Our results highlight substantial virulence potentials displayed by typical African *H. pylori* isolate, including the ability to deregulate carcinogenic pathways in epithelial cells via translocation of a functional CagA oncogene protein. It would therefore be more interesting to assess at the population level, the epidemiological distribution of strains with the similar biological characteristics and which could represent a significant risk of developing gastric cancer in African populations. This will facilitate a better understanding of the risk of gastric cancer in Africa and will contribute to the elucidation of the so-called African Enigma, which still refers, according to Agha A. et al. [14], to an epidemiological situation warranting further clarification. The message in this report does not establish strain KE21 as the cause of the SRCC diagnosed in our patient, but it is more a call for increased surveillance efforts and enhanced research, including genomic explorations, regarding *H. pylori* isolates circulating in Africa and related gastric cancer risk.

## 5. Materials and Methods 

### 5.1. Patient and Biological Sampling

*H. pylori* KE21 was obtained from the gastric mucosa of a Kenyan female patient who underwent gastro-duodenal endoscopy at Aga Khan University Hospital, Nairobi. This strain was isolated through culture of two gastric biopsy specimens sampled from the gastric antrum and corpus of the patient. The culture process was performed by homogenizing the gastric biopsy specimen and inoculating on Brucella agar (BD Difco, USA) supplemented with 7% sheep blood. The culture plates were incubated under microaerophilic conditions (10% CO_2_, 5% O_2,_ and 85% N_2_) at 37 °C for up to 7 days. *H. pylori*-like colonies with translucent, convex morphology grew on the plates and were identified based on biochemical properties (catalase, oxidase, and urease reactions) and microscopic morphology following a Gram staining (Gram negative bacilli). Then, they were sub-cultured for 72 h before being stored at −80 °C in a Brucella broth medium containing glycerol, until shipped in cold-chain to Oita University in Japan where the genomic sequencing was performed. In addition, two biopsy specimens were sampled from the stomach, fixed in 10% buffered formalin, and embedded in paraffin for histological examination by a clinical pathologist. 

### 5.2. Antimicrobial Susceptibility Testing

The antimicrobial susceptibility was assessed using the E-Test^®^ (bioMérieux) method on culture growth of *H. pylori* colonies isolated from gastric biopsy specimens following the Clinical and Laboratory Standards Institute protocols (Wayne, PA, USA). *H. pylori* culture was suspended at a turbidity equivalent to a 3.0 McFarland standard and inoculated onto Müeller–Hinton agar plates supplemented with 7% sheep blood and antibiotics (AMX, CLA, LEVO, TET and MTZ). The MICs of antibiotics were determined after 72 h of incubation. The *H. pylori* strain 26695 was used as a control strain. Clinical breakpoints between resistant and susceptible strains were determined following the guidelines of the European Committee on Antimicrobial Susceptibility Testing (EUCAST) available at http://www.eucast.org/. 

### 5.3. DNA Extraction, Library Preparation, and Whole Genome Sequencing

Harvested confluent bacterial cultures expanded from a single colony of *H. pylori* KE21 isolate were used for the extraction of total DNA by DNeasy Blood and Tissue kit (QIAGEN Inc., Valencia, CA, USA). 

#### 5.3.1. Short-Read Illumina Sequencing

A library of 1 ng DNA was prepared for sequencing of 300-bp paired-end reads, using the Nextera XT DNA Library Preparation kit (Illumina, San Diego, CA, USA). Whole-genome sequencing was performed at 300 cycles using the Illumina Miseq platform (Illumina, Inc., San Diego, CA, USA) following the Manufacturer’s instructions. Fluorescent images were assessed with the MiSeq Control Software, and FASTQ-formatted sequence data were created with MiSeq Reporter Analysis Software. The density cluster and Q-score ≥ 30 of sequenced reads reached 1206 k/mm^3^ and 88%, attesting the good quality of sequencing runs. 

#### 5.3.2. Long-Read MinION Sequencing.

To produce long-read sequences of this strain, we applied 400 ng of genomic DNA on the Oxford Nanopore MinION (Oxford Nanopore Technologies, Oxford, UK) device following the Rapid Sequencing protocol (SQK-RAD004). Raw sequence reads were uploaded to the Epi2Me interface (Metrichor, Oxford, UK) for base calling and demultiplexing of MinION data. Epi2Me was used also for examining basic metrics of sequencing abundance and quality. Only base-called data passing Epi2Me quality parameters (q_mean_ > 6) were downloaded off the cloud in FAST5 and FASTQ formats to use in further analyses. In total, 13,386 MinION reads were obtained with average length and quality score of 6963 bp and 8.3, respectively. 

### 5.4. Bioinformatic Analyses

Low-quality bases (the quality of bases < Q30) and adapters were trimmed using Trimmomatic v0.30 [80]. Filtered High-throughput short reads were screened for the presence of known plasmids using the PlasmidSeeker tool with 26695 (NC_000915.1) as the reference genome [81]. MinION long reads were trimmed using Porechop v.0.2.4 (https://github.com/rrwick/Porechop), assembled along with Illumina Miseq short reads in a hybrid genome assembly using Unicycler v.0.4.8 [82]. The draft genome was polished by using Pilon v.1.23 [83]. The quality assessment of the obtained genome was assessed using QUAST v5.0.2 [84]. The taxonomic assignment of the genome was performed using the WIMP workflow of Epi2Me [17] and the FastANI [18] through the DFAST Quality Control tool of DFAST v1.1.5 (https://dfast.nig.ac.jp/, Tokyo, Japan, 2020). Gene sequences were annotated with Prokka v1.14.5 [85] and DFAST v1.1.5 (https://dfast.nig.ac.jp/). They were functionally characterized and clustered in subsystems by the RASTtk pipeline of Rapid Annotation using Subsystem Technology v2.0 [86]. 

Additionally, 14 *H. pylori* genomes publicly available that had been previously well-characterized, were used along with strain KE21 to infer the pan-genome and its core genome by using Roary v3.13.0 with an 80% BLASTp percentage identity cut-off [87]. The phylogenetic characterization of the strain KE21 was estimated through a bootstrapped neighbor-joining tree estimated by Maximum Likelihood method with MEGA v7 [88] based on the core genome alignment. MAUVE was used for comparing the genome of KE21 with those of J99 and 26695, two reference isolates [50,81]. Nucleotide sequences and inferred amino acid sequences were aligned with references and visually analyzed using CLC genomic Workbench v8.5.1 and in MEGA v7. Maps of single genome and genomes alignment were constructed using CGView Sever v1.0 [89]. ABRicate v1.0.1 (https://github.com/tseemann/abricate) was applied to construct the virulome of *H. pylori* KE21 by detecting putative virulence factors in silico through a BLAST+ against a reference database locally optimized for *H. pylori* species by including additional species-specific virulence genes to the VFDB repository of bacterial virulence factors from various pathogens (http://www.mgc.ac.cn/VFs/) [90]. The *H. pylori* virulome database included 167 non-redundant genes retrieved from *H. pylori* J99 and 26695 isolates. 

### 5.5. AGS Cell Line Co-Infection with H. pylori KE21

The virulence ability of *H. pylori* KE21 strain was further assessed experimentally by infecting human gastric epithelial AGS cell lines as described previously [91]. The experiments were performed twice independently in triplicate. Briefly, AGS cells were seeded into 6-well plates and grown overnight in RPMI 1640 medium supplemented with 10% FBS. The plates were incubated at 37°C for the indicated periods of time in a humidified environment containing 5% CO_2_ and 95% air. K21 strain was harvested from an agar dish and washed twice with PBS before being added to the AGS culture wells with a bacterium-to-cell ratio of 50:1. After 24 h of co-culture, formation of the hummingbird phenotype was examined microscopically in ten randomly chosen fields. Additionally, the functionality of the cagPAI was assessed through measurement of the concentration of induced IL-8 in the supernatant of AGS cells co-cultured with *H. pylori* by using the CXCL8/IL-8 ELISA Kit (R & D Systems, Minneapolis, MN, USA). 

### 5.6. Nucleotide Sequence Accession Number

The genome sequenced of *H. pylori* KE21 was deposited at the DNA Data Bank of Japan (DDBJ) under the accession number AP023320. 

### 5.7. Ethical Consideration

The patient KE21 gave informed consent for the conduct of this study, in accordance with the Declaration of Helsinki. The study was approved by the Institutional Ethics Committee of the Aga Khan University Hospital (Ref N#: 2017/REC-97(vl)), the Kenyatta University Ethical Review Committee (Ref N# PKU/509/1602-PKU/447/E39), and the Oita University Ethical Review Committee (Ref N#: 1660). 

## Figures and Tables

**Table 1:** Main virulence factors encoded in the genome of *H. pylori* KE21*.

Virulence Factor	Gene	Start	End	% Coverage	% Identity	Characteristics
CagA	hp0547	750,676	754,221	99.49	94.58	1 copy
VacA	hp0887	1,114,652	1,118,533	99.69	91.78	s1m1
HtrA	hp1018	639,400	640,830	99.93	95.53	1 copy
CGT	hp0421	1,272,696	1,273,861	99.66	95.20	1 copy
GGT	hp1118	1,371,288	1,372,991	100.00	95.31	1 copy
DupA	hp0441	−	−	−	−	Absent
IceA	hp1209	−	−	−	−	Absent
OipA/HopH	hp0638	852,806	853,731	99.89	93.85	1 copy in “ON” status
AlpA/HopC	hp0912	1,143,117	1,144,676	100.00	94.49	1 copy
AlpB/HopB	hp0913	1,144,698	1,146,287	99.81	94.35	1 copy
HopQ/Omp27	hp1177	1,434,514	1,436,428	99.17	88.18	1 copy hopQ type1 allele
BabA/HopS	hp1243	1,124,618; 1,507,238	1,126,840; 1,509,404	99.19; 96.69	87.64; 87.32	2 copies
BabB/HopT	hp0896	−	−	−	−	Absent
BabC/HopU	hp0317	−	−	−	−	Absent
HomA	hp0710	937,597; 1,173,611	93,9589; 1,175,576	98.03; 98.29	85.96; 90.64	2 copies
SabA/Omp17	hp0725	952,657	954,511	97.99	89.10	1 copy
SabB/HopO	hp0722	948,177	949,975	98.30	88.06	1 copy

(*) Gene: the gene name as in 26695 reference isolate; Start: start nucleotide-position in the genome of strain KE21; End: the end nucleotide-position in the genome of strain KE21; % Coverage, the coverage of the query blast against the reference sequence; % Identity, the proportion of nucleotide matching between the query sequence in strain KE21 and the reference sequence.

## Supplementary Materials

The following are available online at https://www.mdpi.com/2072-6651/12/9/556/s1, Figure S1: N-terminal sequence alignment of genes encoding the Outer inflammatory protein A (OipA) of *H. pylori* KE21 and reference strains, Figure S2: Molecular Phylogenetic analysis of genes encoding the Helicobacter outer membrane protein Q (HopQ) of KE21 and reference strains by Maximum Likelihood method, Table S1: Summary statistics of the pan-genome between *H. pylori* strains KE21, J99, and 26695, Table S2: The pan-genome of orthologous genes formed by KE21, J99, and 26695 strains, Table S3: Baseline characteristics of genomes used in this study, Table S4: Characterization of the transposon plasticity zone (TnPZ) and the integrating conjugative element (ICE*Hptfs*) identified in *H. pylori* KE21, Table S5: Predicted dg-region and *cag* pathogenicity island (cagPAI) in *H. pylori* KE21, Table S6: The virulome of *H. pylori* KE21.
