# Supplementary material for: Whole Genome Sequencing Reveals Virulence Potentials of Helicobacter pylori Strain KE21 Isolated from a Kenyan Patient with Gastric Signet Ring Cell Carcinoma
Source: Toxins (Basel). 2020 Aug 29;12(9):556. doi: 10.3390/toxins12090556 (PMC7551074; doi:10.3390/toxins12090556)
Supplement: Supplementary file 1 [file toxins-12-00556-s001.zip › Figure S1_toxins-887356.pptx]

## Slide 1
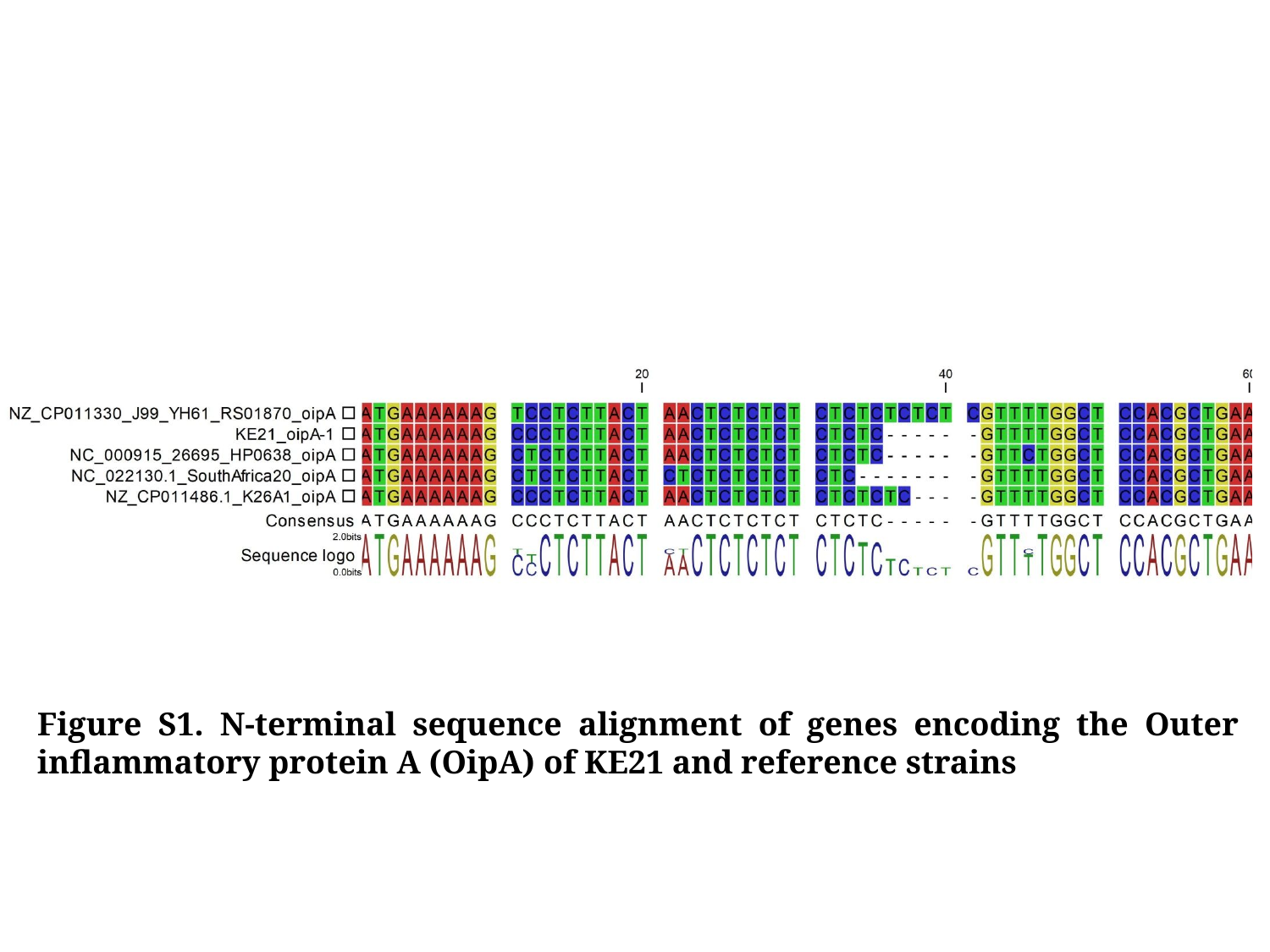

Figure S1. N-terminal sequence alignment of genes encoding the Outer inflammatory protein A (OipA) of KE21 and reference strains
